# Inequity under equality: research on the benefits equity of Chinese basic medical insurance

**DOI:** 10.1186/s12913-020-05584-w

**Published:** 2020-08-03

**Authors:** Li Diao, Yiwei Liu

**Affiliations:** 1 Center for Social Security Studies, Wuhan University, 16 Bayi Road, Wuchang District, Wuhan, 430072 China; 2 School of Government, Central University of Finance and Economics, 39 South College Road, Haidian District, Beijing, 100081 China

**Keywords:** Medical insurance, Benefits equity, Different income groups, China

## Abstract

**Background:**

The pursuit of equity is one of the basic principles behind the strengthening of health care reform. China’s new rural cooperative medical insurance (NRCMI) and urban residents’ basic medical insurance (URBMI) are both “equalized” in terms of fundraising and reimbursement. This paper studies the benefits equity under this “equalized” system.

**Methods:**

The data analysed in this paper are from the China Family Panel Studies (CFPS) from 2014 to 2016, implemented by the Institute of Social Science Survey at Peking University. A two-part model and a binary choice model are used in the empirical test.

**Results:**

The empirical test revealed that high-income people benefit more from basic medical insurance than low-income people. Mechanism analysis demonstrated that high-income people have higher medical insurance applicability and can utilize better health care. Since low-income people are unhealthier, inequity in benefits exacerbates health inequity. We also found that the benefits equity of URBMI is better than that of NRCMI.

**Conclusions:**

The government needs to pay more attention to the issue of medical insurance inequity. We should consider allowing different income groups to pay different premiums according to their medical expenses or applying different reimbursement policies for different income groups.

## Background

Since the 1990s, addressing health inequities and improving the health of vulnerable groups have been the core goals of health care reform in many countries [1]. Governments continue to improve the medical insurance system and increase the supply of medical services to improve medical insurance coverage and accessibility among vulnerable groups and reduce their personal burdens related to medical services [2]. However, a study by the World Bank [3] found that increased health insurance coverage does not promote health equity, as it is not poor individuals who benefit more from health insurance. Based on transnational data, Davoodi et al. [4] and Wagstaff et al. [5] reached similar conclusions. As in most other countries, the Chinese government has attached great importance to health insurance reform in the past decade and aims to improve the fairness of medical insurance and accessibility to medical services. The realization of residents’ health equality is an important goal of health care reform [6, 7].

At present, China has implemented urban residents’ basic medical insurance (URBMI) and new rural cooperative medical insurance (NRCMI) for urban and rural residents, respectively. These two medical insurance systems are “equalized” in terms of fundraising and reimbursement. Specifically, in the same designated area, each insured person pays the same premium on average and has the same reimbursement percentage. URBMI and NRCMI are designed to guarantee that all insured individuals have “equal opportunity” [8]. However, equal opportunity does not mean “equal results”. According to the system design, individuals with higher health care utilization can receive more reimbursement, and such differences in health care utilization will lead to differences in the associated benefits [9]. The goal of basic medical insurance is to ensure that every citizen has access to medical services, with a focus on providing low-income people with better health care [10]. Thus, while reducing the financial burden of medical care for all people, whether basic medical insurance can provide more benefits to low-income people has become an important issue that deserves attention.

From the perspective of social equity and justice [11, 12], vulnerable groups should be the focus of social medical insurance [13]. This focus should be not only the basic principle of China’s health care system reform but also the value orientation of China’s social security system. Therefore, assessing the fairness of the actual benefits of the insured population at different income levels will help policy makers enhance medical insurance policies [14]. The Chinese government has continuously increased its financial subsidization of basic medical insurance to increase benefits and promote social equity [15]. In 2019, the financial subsidy standards for URBMI and NRCMI were raised from 490 yuan to 520 yuan per person per year. However, if the basic medical insurance system cannot effectively provide more benefits to low-income people, then the corresponding financial subsidy will play a role in further promoting the inequity of health care utilization.

Equity is an important indicator for evaluating the effectiveness of health care reform [16]. According to the World Health Organization’s ranking of health care system financing equity and distribution justice among its member states, China ranks fourth from the bottom [17]. After decades of health care system reform, especially the “healthy China” strategy proposed by the Chinese government, how has the equity of the basic medical insurance system improved? Mathematical methods and representative data are needed for evaluation. To address this question, this paper uses China Family Panel Studies (CFPS) data from 2014 to 2016. CFPS is implemented by the Institute of Social Science Survey at Peking University. A total of 46,166 samples were analysed with a two-part model and binary choice model in this paper. Does the “equal opportunity” design of the medical insurance system achieve equal benefits for participants with different incomes? If not, do low-income people benefit less than high-income people? We also examined whether there is a difference in benefits equity between URBMI and NRCMI.

### Literature review

Many scholars have studied the equity of medical insurance and public health services. Doorslaer et al. [18] compared the correlation between income and health inequity in nine developed countries and found that the correlation between the income and health of American and British residents is stronger than that in other developed countries, indicating higher health inequity. Pannarunothai and Mills [19] studied health care utilization and medical insurance equity among urban residents in Thailand. The study showed that there was no significant difference in hospitalization rate among different income groups but that the lowest income group had the lowest reimbursement rate. Gwatkin et al. [20] discussed how to promote the equity of the health care system and suggested that direct compensation for poor or vulnerable people is an effective approach. Chu et al. [21] evaluated the implementation effect of National Health Insurance (NHI) in Taiwan, China, by comparing out-of-pocket payments before (1994) and after (1996) the implementation of the insurance system. They found that NHI narrowed the gap in out-of-pocket payments for families with different economic conditions and promoted health care equity. Castro-Leal et al. [22] examined the status of fiscal subsidies to public health in seven African countries and found that these subsidies benefit rich people more than poor people.

Since the 1990s, with the improvement of China’s medical system and the increase in the supply of medical services, many scholars have analysed the equity of public health services in China. Most of the early literature focussed on the macro or meso level. For example, by studying the medical financing mechanism for rural areas, Ping [23] noted that poorer farmers had more financial burden than their richer counterparts. According to Wang et al’s [24] research based on national health service survey data, China’s market-oriented medical reform has created inequity in health care service utilization. Using regional statistical data, Wei and He [25] pointed out that such inequity exists not only between but also within urban and rural areas. Feng and Chen [26] reported that the main beneficiaries of public medical services are residents in high-income areas.

In recent years, research using household survey data has become mainstream. Compared with macro- or meso-level data, household survey data allow basic medical insurance benefits and socioeconomic characteristics to be directly observed. Examining information on medical insurance participants and public medical service beneficiaries, some scholars have concluded that China’s basic medical insurance is inequitable for individuals with different income levels. For example, Xie E [27] and Qi and Li [28], based on the China Health and Nutrition Survey (CHNS) data, found that China’s basic medical insurance benefits and public health service utilization obviously favour the wealthy [29]. Zhou et al. [30] found that income is the main determinant of inpatient service utilization based on China’s 2003 and 2008 National Health Service Survey (NHSS) data. Based on data from household surveys in 10 western provinces in China conducted in 2005, Liu et al. [31] drew similar conclusions. In addition, out-of-pocket payments clearly do not favour poor people. According to Doorslaer et al. [32], based on household survey data from 14 Asian countries and regions, out-of-pocket payments are heavily biased against poor people, which increases poverty rates and aggravates poverty depth in China. Wang [33] found that inequity still exists in rural areas, even after the integration of urban and rural medical insurance, and that there is still a gap between the actual and expected outcomes of URBMI.

More specifically, some scholars have focused their research on the equity of NRCMI and URBMI. For example, Li [34] analysed whether NRCMI can alleviate inequity and found that the design of the rural cooperative medical insurance financing system has not produced vertical equity. Shen et al. [35] examined the equity of NRCMI and found considerable inequity in individual payments. Wong et al. [36] used Wuhan city as an example to discuss the reform of China’s urban medical system; the reform is believed to have had some effect, but the benefit for vulnerable people is small. Zhou et al. [9] conducted a study of urban residents in China and found that the level of medical insurance compensation for low-income people was significantly lower than that for high-income people.

In summary, regarding equity in the health care system, valuable efforts have been made by previous studies. Following these previous studies, we tested whether URBMI and NRCMI, which are equalized in terms of fundraising and reimbursement, can benefit the insured equally and if these two systems are equitable themselves. We also used two-part models to reduce the estimated bias caused by the endogeneity and sample selection bias that may exist in the model, ensuring the accuracy of the results.

## Methods

### Data source

This paper analysed data from CFPS obtained from the Peking University Open Research database. Compared to other micro databases, CFPS has more comprehensive information on health care utilization. The survey was conducted in 19,986 households in 986 villages/communities of 162 districts/counties in 25 provinces/cities/autonomous regions across China, and all economically linked family members in these households were targeted. The 2010 baseline survey included a total of 14,960 households and 57,155 baseline respondents, including 33,600 adults aged 16 years and older and 8990 children aged 15 years and younger. Panel surveys were conducted in 2012, 2014 and 2016. Among these surveys, the 2012 panel survey successfully accessed 42,970 individuals in 12,725 households; the second panel survey in 2014 successfully accessed 45,738 individuals in 14,237 households; and the third panel survey in 2016 successfully accessed 41,761 individuals in 14,810 households.

Since we focused on the issue of the equity of basic medical insurance benefits after the “new health care reform” in China, data from the latest survey years, 2014 and 2016, were merged into pooled cross-sectional data to conduct an empirical analysis. A total of 87,499 insured samples were included in the original data. After using the traditional casewise method to drop samples with missing information, a total of 46,166 samples were analysed in the study. It should be noted that in our initial sample, sample loss was mainly due to the lack of hospitalization information in the dataset. We analysed the distribution of the missing samples and found that each county had approximately 50–60 sample losses, all of which were random, not subjective, losses. Notably, among these samples, 3810 respondents experienced hospitalization. In addition, we also studied the health status of the insured population (including the incidence of hospitalization, the incidence of chronic diseases and self-reported health (SRH)) and health care utilization.

### Variables

1. Dependent variable. First, we used hospitalization expense reimbursement to analyse medical insurance benefits. Hospitalization expense reimbursement is a continuous variable and was log-transformed prior to analysis.

Next, we analysed whether the difference in benefits is due to differences in the health status of different income groups. In this paper, sick in the last 2 weeks, sick in the last half year and SRH are the three proxy variables of health status. Among them, if the respondent became sick in the last 2 weeks, the value of “sick in the last 2 weeks” is 1; otherwise, it is 0. If the respondent became sick in the last half year, the value of “sick in the last half year” is 1; otherwise, it is 0. The SRH data were processed in this study. If the answer to “What is your health status?” is “Fair, good, excellent or perfect”, then the value is 0; if the answer is “Poor”, then the value is 1.

2. Independent variable. The independent variable in this paper is family income. Referring to the study of Wagstaff [16], we divided household income from low to high into five groups. The number of samples in each group is the same, and the assignments are 1, 2, 3, 4 and 5.

3. Mechanism variables. After excluding or controlling the effects of health status, we examined the causes of inequity in medical insurance benefits. In the model, the variables “medical insurance applicability”, “total hospitalization expense” and “medical institution choice” are analysed. Among them, “medical insurance applicability” is determined by the following question: “After medical treatment, whether it is because of the payment line, location restriction, the coverage of medical insurance reimbursement, etc., will your medical insurance pay for your medical expenses?”; if the answer is yes, the value is 1, and otherwise, it is 0. “Medical institution choice” is determined by the following question: “What kind of medical institution do you usually choose when you are sick?”; if the respondent chooses a general hospital or specialist hospital, then the value is 1, and if the respondent chooses a community health service centre, township hospital or clinic, then the value is 0.

4. Control variables. To accurately estimate the equity of medical insurance benefits, we added the individual and family characteristic variables of the insured group to the regression analysis. These variables mainly include sex (dummy variable: male = 1, female = 0), age (continuous variable), age squared, marital status (dummy variable: married = 1, unmarried = 0), years of schooling (continuous variable), and family size (continuous variable).

### Model construction

#### 1. Two-part model

When analysing the differences in the medical insurance benefits of different income groups, there are too many respondents with no medical insurance reimbursement. There are several reasons for this. One is that because of the existence of a minimum deduction, there is a discrepancy between the amount the patient expects to be reimbursed and the amount reimbursed. Another is that some insured people may choose not to be hospitalized due to reasons such as their health status or their own medical insurance system; in such cases, no hospitalization expenses were incurred, and there was therefore no opportunity to get reimbursed. In addition, complex reimbursement procedures can also cause patients to lose their reimbursement. These factors result in the non-normal distribution of sample errors and introduce bias into the OLS estimation. A two-part model proposed by Duan et al. [37] was used to solve this problem; it comprised a selection model and an outcome model. In this study, the probit model is used as the selection model:

1 $$ pr\left(R{\mathrm{eimburse}}_{\mathrm{m}}=1\mathrm{Income},\mathrm{X}\right)=\theta \left({\alpha}_0+{\sum}_{n=1}^5{\alpha}_n\ast {Income}_{mn}+X{}_m\kappa +{e}_m\right) $$

where *θ* (·) is the standard normal cumulative distribution function and *R*eimburse_m_ refers to the dichotomous variable of whether the medical insurance reimbursement amount is over 0. If the medical insurance reimbursement amount for hospitalization expenses is over 0, then the value is 1; otherwise, it is 0. *Income*_*mn*_ is the income of respondent m in group n, where n is a value of 1, 2, 3, 4, or 5; *X*_*m*_ is a series of control variables; and *e*_*m*_ is a random disturbance.

The general linear model (GLM) is used as an outcome model to estimate non-zero medical insurance reimbursement:

2 $$ Log\left(R{\mathrm{eimburse}}_{\mathrm{m}}\right)={\alpha}_0+{\sum}_{n=1}^5{\alpha}_n\ast {Income}_{mn}+\beta {}_m\kappa +{e}_i $$

All of the respondents’ medical insurance reimbursements analysed in this model were over 0. The random disturbances *e*_*m*_ and e_*i*_ of eqs. (1) and (2) are assumed to be unrelated; that is, the medical insurance reimbursement amounts of 0 and over 0 are independent of each other. *β*_*m*_ is a series of control variables that may affect medical insurance reimbursement, and e_*i*_ is the residual term.

#### 2. Binary choice model

The binary choice model is used to estimate the binary discrete dependent variable. The model is used in this paper to analyse the influencing factors of health status, hospitalization choice and medical insurance applicability. The regression equation of hospitalization choice is the same as eq. (1) and will not be described here. The probit models for health status and medical insurance applicability are as follows:

3 $$ pr\left({Health}_{\mathrm{m}}=1\mathrm{Income},\mathrm{X}\right)=\theta \left({\alpha}_0+{\sum}_{n=1}^5{\phi}_n\ast {Income}_{mn}+X{}_m\kappa +{\chi}_m\right) $$

4 $$ pr\left(R{\mathrm{eimburse}}_{\mathrm{j}}=1\mathrm{Income},\mathrm{X}\right)=\theta \left({\alpha}_0+{\sum}_{n=1}^5{\phi}_n\ast {Income}_{mn}+X{}_m\kappa +{\varpi}_m\right). $$

Similarly, *θ* (·) is a standard normal distribution function, assuming that the random perturbation terms *X*_*m*_ and *ϖ*_*m*_ follow a standard normal distribution, where *Health*_m_ denotes the dummy variables “sick in the last 2 weeks”, “sick in the last half year”, and “SRH”. *R*eimburse_*j*_ refers to medical insurance applicability, and the control variable *X*_*m*_ is the same as in eq. (1).

In addition, some respondents were repeatedly counted in the study. To improve the reliability of the significance test, all regression coefficient standard errors were cluster corrected at the individual level.

## Results

### Descriptive analysis of the variables

STATA 15 was the main tool used for data analysis in this paper. The descriptive analysis of the variables is shown in Table 1. A total of 12.62% of the respondents with URBMI were hospitalized, while 11.68% of the respondents with NRCMI were hospitalized. The average reimbursement amount of URBMI was much higher than that of NRCMI. In the last 2 weeks, 28.81% of the respondents with URBMI were sick, while the percentage for those with NRCMI was 29.36%; in the last half year, 19.55% of the respondents with URBMI were sick, while the percentage for those with NRCMI was 16.17%. A total of 85.96% of the respondents with URBMI rated their health status as “Fair, good, excellent or perfect”, while the percentage of the respondents with NRCMI who did was 83.56%. In addition, 69.75% of the respondents with URBMI and 59.82% of the respondents with NRCMI can be reimbursed by their medical insurance. The average hospitalization expenses of respondents with URBMI totalled 11,882.75 yuan, while the average hospitalization expenses of respondents with NRCMI totalled 17,272.32 yuan. More respondents with URBMI than with NRCMI chose to receive health care services in general hospitals or specialist hospitals. In addition, concerning the control variables, respondents with URBMI and NRCMI had the same characteristics in the following respects: the average age for both groups was approximately 46 years old; there were proportionally more males than females in both samples; and over 70% of respondents were married in both groups. The average years of schooling of respondents with URBMI was 8.50 years, 1.97 years higher than that of respondents with NRCMI. Compared with that of respondents with NRCMI, the family size of respondents with URBMI was smaller.

**Table 1:** Descriptive analysis of the variables

Variables	URBMI		NRCMI	
	Percentage	Mean	Percentage	Mean
Hospitalization choice	No = 87.38%, Yes = 12.62%		No = 88.32%, Yes = 11.68%	
Hospitalization expense (CNY) reimbursement		11,295.78 (22,968.99)		6227.14 (13,361.58)
Sick in the last 2 weeks	No = 71.19%, Yes = 28.81%		No = 70.64%, Yes = 29.36%	
Sick in the last half year	No = 80.45%, Yes = 19.55%		No = 83.83%, Yes = 16.17%	
SRH	Fair, good, excellent or perfect = 85.96%, Poor = 14.04%		Fair, good, excellent or perfect =83.56%, Poor = 16.44%	
Medical insurance applicability	No = 30.25%, Yes = 69.75%		No = 40.18%, Yes = 59.82%	
Total hospitalization expenses (CNY)		11,882.75 (22,914.01)		17,272.32 (32,290.44)
Medical institution choice	General hospital or specialist hospital =38.51%, community health service centre, township hospital or clinic =61.49%		General hospital or specialist hospital =71.00%, community health service centre, township hospital or clinic =29.00%	
Household income	Poorest = 9.88%, 2nd = 9.18%, 3rd = 18.71%, 4th = 28.24%, Richest = 33.98%		Poorest = 24.78%, 2nd = 15.35%, 3rd = 23.96%, 4th = 22.75%, Richest = 13.17%	
Age		46.15 (16.65)		46.30 (17.73)
Age squared		2407.28 (1583.11)		2458.69 (1691.76)
Sex	Female = 55.74%, Male = 44.26%		Female = 50.94%, Male = 49.06%	
Marital status	Unmarried = 27.16%, Married = 72.84%		Unmarried = 19.33%, Married = 80.67%	
Years of schooling		8.50 (4.66)		5.53 (4.44)
Family size		3.81 (1.73)		4.51 (2.15)

Note: Percentage of each value of all dummy variables and mean of all continuous variables are shown in the table. Numbers in parentheses are standard deviations. According to National Bureau of Statistics of China, average exchange rate of CNY against USD in 2016 is 6.6423

#### Descriptive analysis

Table 2 shows a significant difference in medical service utilization among different income groups. First, in terms of respondents with URBMI, total hospitalization expenses and income were positively correlated, with respondents in the lowest income group spending an average of 14,188.38 CNY/year and those in the highest income group spending 22,722.83 CNY/year. The same conclusion holds for NRCMI, with the lowest income group spending 10,335.58 CNY/year and the highest income group spending 12,637.06 CNY/year. Second, medical insurance reimbursement was positively correlated with both income and total hospitalization expenses. The reason for this may be that groups with higher income utilize more and higher-quality health care. Third, in both the URBMI and NRCMI samples, the medical insurance applicability of the lower income groups was worse, which may increase the inequity of medical insurance benefits.

**Table 2:** Health care utilization among insured individuals

Quintile	URBMI			NRCMI		
	Total hospitalization expenses (CNY)	Hospitalization expense reimbursement (CNY)	Medical insurance applicability	Total hospitalization expenses (CNY)	Hospitalization expense reimbursement (CNY)	Medical insurance applicability
Poorest	14,188.38	6005.48	0.63	10,335.58	4613.19	0.59
	(15,231.84)	(5710.46)	(0.48)	(23,549.75)	(9336.36)	(0.49)
2nd	14,618.51	5625.16	0.67	11,467.72	4570.25	0.60
	(17,658.92)	(9493.36)	(0.47)	(19,925.77)	(14,682.43)	(0.49)
3rd	15,301.49	7320.51	0.70	12,303.68	4617.85	0.60
	(20,622.77)	(10,958.06)	(0.46)	(25,200.23)	(9347.92)	(0.49)
4th	15,659.26	9869.67	0.72	12,583.4	5836.23	0.60
	(34,028.64)	(18,342.27)	(0.45)	(21,770.66)	(12,320.78)	(0.49)
Richest	22,722.83	15,899.01	0.81	12,637.06	6598.45	0.62
	(45,446.14)	(35,098.55)	(0.40)	(18,804.46)	(14,800.75)	(0.49)
*P* value (between the poorest group and the richest group)	***	***	***	***	***	***

Note: numbers in parentheses are standard deviations; ***, **, and * indicate significance levels of 1, 5, and 10%, respectively; the T-test method is used to test the health care utilization of the richest and poorest groups. According to National Bureau of Statistics of China, average exchange rate of CNY against USD in 2016 is 6.6423

If the positive correlations among medical insurance reimbursement, health care utilization and income are because people with higher incomes are unhealthier, then the conclusion that there is inequity in medical insurance benefits cannot be drawn. Table 3 shows the health status of the insured populations at different income levels; sick in the last 2 weeks, sick in the last half year and SRH are used to measure health status. In the sample of respondents with URBMI, from the lowest income group to the highest income group, the 2-week sickness rate dropped from 24 to 19%, and the half-year sickness rate dropped from 43 to 26%. The percentage of people with poor SRH dropped from 26 to 10%. In the sample of respondents with NRCMI, from the lowest income group to the highest income group, the 2-week sickness rate dropped from 20 to 13%, and the half-year sickness rate dropped from 36 to 25%. The percentage of people with poor SRH dropped from 25 to 10%. People with higher incomes are therefore healthier than their lower-income counterparts. Therefore, the assumption that high-income people receive more medical services due to poor health is not confirmed. Of course, to scientifically verify this conclusion, an empirical test is still needed.

**Table 3:** Health status of insured individuals

Quintile	URBMI			NRCMI		
	Sick in the last two weeks	Sick in the last half year	SRH	Sick in the last two weeks	Sick in the last half year	SRH
Poorest	0.24	0.43	0.26	0.20	0.36	0.25
	(0.42)	(0.50)	(0.44)	(0.40)	(0.48)	(0.43)
2nd	0.23	0.30	0.22	0.18	0.30	0.19
	(0.42)	(0.46)	(0.42)	(0.38)	(0.46)	(0.39)
3rd	0.19	0.31	0.17	0.16	0.28	0.15
	(0.39)	(0.46)	(0.37)	(0.37)	(0.45)	(0.36)
4th	0.19	0.26	0.11	0.13	0.25	0.11
	(0.39)	(0.44)	(0.31)	(0.34)	(0.44)	(0.32)
Richest	0.19	0.26	0.10	0.13	0.25	0.09
	(0.39)	(0.44)	(0.30)	(0.33)	(0.43)	(0.29)
P value (between the poorest group and the richest group)	***	***	***	***	***	***

Note: numbers in parentheses are standard deviations; ***, **, and * indicate significance levels of 1, 5, and 10%, respectively; the T-test method is used to test the health care utilization of the richest and poorest groups

#### Empirical test

##### 1. Test of the equity of medical insurance benefits

Table 4 reports the differences in medical insurance reimbursement for different income groups. Among respondents with URBMI, we found that there was no significant difference between the lowest income group and the second- and third-lowest income groups; however, the reimbursement rates in the fourth-lowest income group and the highest group are approximately 8.95 and 12.7% higher than that in the lowest group, respectively. Among respondents with NRCMI, the reimbursement rates of the second-, third-, and fourth-lowest income groups and the highest-income group were approximately 3.12, 3.77, 5.87 and 5.98% higher than that of the lowest income group, respectively. We also compared the differences between URBMI and NRCMI and found that the benefits equity of URBMI is better than that of NRCMI. The reason for this may be that the income gap in rural areas is wider than that in urban areas.

**Table 4:** Impact of income on medical insurance reimbursement

Variable	URBMI		NRCMI	
	Selection model (probit)	Outcome model (GLM)	Selection model (probit)	Outcome model (GLM)
Reference group: Poorest				
2nd	0.0917	0.0122	0.075	0.0312**
	(0.280)	(0.039)	(0.073)	(0.015)
3rd	0.102	0.0164	0.024	0.0377***
	(0.282)	(0.036)	(0.079)	(0.012)
4th	0.561*	0.0895**	0.079	0.0587***
	(0.295)	(0.035)	(0.070)	(0.011)
Richest	0.658**	0.127***	0.128*	0.0598***
	(0.286)	(0.036)	(0.073)	(0.012)
Control variable	YES	YES	YES	YES
Constant	1.456*	0.446***	0.309	0.366***
	(0.775)	(0.102)	(0.247)	(0.039)
Observations	498	498	3312	3312

Note:Control variables include respondents’ age, age squared, sex, years of schooling, marital status and family size; ***, **, and * indicate significance levels of 1, 5, and 10%, respectively

The descriptive analysis previously revealed that the reason that higher-income people are reimburse more by their medical insurance is not because people in this group are unhealthier, and the probit model will be used to ensure the robustness of this conclusion in this section (see Table 5).

**Table 5:** The impact of income on health

Variable	URBMI			NRCMI		
	Sick in the last two weeks	Sick in the last half year	SRH	Sick in the last two weeks	Sick in the last half year	SRH
Reference group: Poorest						
2nd	−0.291***	−0.115	−0.0415	−0.106***	0.0125	−0.113***
	(0.085)	(0.094)	(0.093)	(0.021)	(0.024)	(0.024)
3rd	−0.274***	−0.109	−0.307***	− 0.116***	0.00871	− 0.210***
	(0.072)	(0.082)	(0.082)	(0.019)	(0.022)	(0.022)
4th	−0.381***	− 0.0105	− 0.503***	−0.165***	− 0.0765***	−0.340***
	(0.069)	(0.078)	(0.080)	(0.024)	(0.023)	(0.024)
Richest	−0.388***	−0.0291	− 0.515***	−0.167***	− 0.110***	−0.459***
	(0.069)	(0.078)	(0.080)	(0.020)	(0.028)	(0.030)
Control variables	YES	YES	YES	YES	YES	YES
Constant	−0.542***	−2.104***	−2.433***	−1.006***	−2.711***	−2.825***
	(0.165)	(0.204)	(0.241)	(0.060)	(0.081)	(0.088)
Observations	4914	4914	4914	41,346	41,342	41,348

Note:Control variables include respondents’ age, age squared, sex, years of schooling, marital status and family size; ***, **, and * indicate significance levels of 1, 5, and 10%, respectively

Table 5 shows that among respondents with URBMI, the incidence of being sick in the last 2 weeks is significantly negatively correlated with income, but there is no significant correlation between the incidence of being sick in the last half year and income. Compared with the lowest income group, the highest-income group and the 4th-lowest income group have better health. Among respondents with NRCMI, the incidence of being sick in the last 2 weeks or half year and SRH were all significantly correlated with income. There was a significant positive correlation between income and health. In fact, according to the definition of equity, respondents with poorer health deserve more medical insurance compensation; therefore, the results indicate that equalized fundraising and reimbursement cannot guarantee the equity of medical insurance benefits and may even deepen health inequities.

Among respondents with URBMI, the lowest income group differed significantly from the second-lowest income group in terms of medical institution choice, but among respondents with NRCMI, this difference was not significant, indicating that more urban residents than rural residents can utilize more expensive and better health care, which leads to more inequitable benefits among respondents with NRCMI.

#### Mechanisms

This section analyses the potential mechanism of inequity in basic medical insurance benefits from three perspectives, and the regression results are shown in Table 6. The highest income group and 4th-lowest income group tended to be hospitalized in general or specialist hospitals at a higher rate than other income groups, and their total hospitalization expenses were significantly higher than those in other groups, indicating differences in health care utilization. In addition, from the perspective of medical insurance applicability, respondents with higher incomes had better medical insurance applicability; although the low-income group is covered by a medical insurance system, they are more likely to receive no reimbursement for their expenses. The reason for this may be that their total expenses do not reach the minimum level required.

**Table 6:** Mechanisms: Total hospitalization expenses, medical insurance applicability and medical institution choice

Variable	URBMI			NRCMI		
	Total hospitalization expenses	Medical institution choice	Medical insurance applicability	Total hospitalization expenses	Medical institution choice	Medical insurance applicability
Reference group: Poorest						
2nd	0.118	0.312***	0.115***	0.0764	0.0272	0.063***
	(0.180)	(0.083)	(0.015)	(0.059)	(0.022)	(0.016)
3rd	0.195	0.230***	0.112***	0.0583	0.0948***	0.081***
	(0.181)	(0.071)	(0.012)	(0.053)	(0.020)	(0.015)
4th	0.486***	0.267***	0.227***	0.194***	0.182***	0.0718***
	(0.180)	(0.067)	(0.015)	(0.059)	(0.020)	(0.016)
Richest	0.495***	0.421***	0.399***	0.213***	0.359***	0.126***
	(0.181)	(0.068)	(0.012)	(0.071)	(0.023)	(0.016)
Control variables	YES	YES	YES	YES	YES	YES
Constant	8.099***	−0.112	−0.335	8.181***	−0.363***	0.407**
	(0.509)	(0.157)	(0.530)	(0.193)	(0.057)	(0.185)
Observations	618	4910	618	4858	41,256	4858

Note: Control variables include respondents’ age, age squared, sex, years of schooling, marital status and family size; ***, **, and * indicate significance levels of 1, 5, and 10%, respectively

## Discussion

Equity is a proposition related to ethics and value judgements and has rich connotations. In the field of economics, scholars have proposed specific definitions for health care equity. For example, Whitehead (1992) defined it as equal medical insurance applicability under the same health care demands, equal medical usage under the same medical needs, and the same quality of health care service for everyone. In 2009, the “Health Care Reform” programme proposed the goal of “total coverage of urban and rural residents with the basic medical insurance system in 2011”. The Chinese government has invested 850 billion CNY over 3 years to expand medical insurance coverage and improve reimbursement standards. Its investment in URBMI and NRCMI accounts for almost 50% of the central government’s medical expenditures. However, in the midst of a renewed call for equity in reform, we cannot ignore the deep inequities that have been brought about by health care reform, such as the inequities in medical insurance benefits and health care utilization due to income.

This paper analyses the issue of benefits equity in Chinese basic medical insurance. If people with different incomes pay the same premiums to the insurance fund and have the same medical needs but obtain different insurance benefits because of the difference in their total medical expenses, then it can be considered that their medical insurance benefits are unfair. We used CFPS data from 2014 and 2016 and found that the reimbursement for high-income people was significantly higher than that for low-income people. However, low-income people are unhealthier and need more health care and expense reimbursement; that is, high-income people are reimbursed more than their premium contribution, while low-income people are reimbursed less. The amount of hospitalization reimbursement in China was calculated based on total hospitalization expenses. Large general or specialist hospitals have better medical services and charge more for medical care; as a result, individuals who obtain care in such institutions easily reach the minimum deduction required for reimbursement. Therefore, the differences in health care utilization and medical insurance applicability are the main causes of inequity in medical insurance benefits.

Moreover, although URBMI and NRCMI are “equalized” in terms of fundraising and reimbursement, NRCMI is less equitable than URBMI in terms of benefits. China has a typical urban-rural dual structure, and the income gaps in urban and rural areas are different. Some scholars have noted that the income gap between individuals with the highest income and those with the lowest income in rural areas is much larger than that in urban areas [38], which means that residents in the highest income group in rural areas are more likely to utilize better health care resources and that residents in the lowest income group are less likely to utilize health care. Thus, NRCMI is more inequitable than URBMI.

Yip, an expert on Chinese health care reform, believes that the greatest challenge facing China’s health care reform is increasing the efficiency of financial input [39]. To achieve the “equity” goal, financial input should bring about better policy outcomes, including improving the equity of medical insurance, the health of residents, the quality of health care services, and patient satisfaction and reducing the financial burden on patients. However, even under an equalized medical insurance system, the equalized medical insurance provided by the government still causes inequity in benefits; that is, low-income people “subsidize” high-income people through their premiums.

Looking back at the reform of China’s health system, it has undergone a process from loss of equity to reshaping equity through an equalized design. In this process, the transformation from policy formulation to policy implementation urgently needs to be realized. Thus, in the design of the medical insurance system, we should consider allowing different income groups to pay different premiums according to their medical expenses or applying different reimbursement policies for different income groups. In addition, broadening and opening up new funding channels and providing more medical assistance for low-income people will prevent difficult situations caused by high medical expenditure [40]. It is also possible to consider increasing the reimbursement rate for primary medical institutions so that low-income people who seek medical care in such institutions will benefit more than if they sought help in other types of institutions.

However, there are some limitations in our analysis. This study used a cross-sectional dataset, which cannot be used to test for a causal relationship between medical insurance and medical insurance reimbursements. In addition, there has been great changes since 2016 in socioeconomic development, life style, consumption attitude, health, or income of residents in China. Using cross sectional data, the present study cannot test those changes. Longitudinal dataset can be used in the future to investigate the causal relationship between medical insurance and medical insurance reimbursements, as well as tracking the changes of either medical insurance or medical insurance reimbursements and testing their relationship over time.

## Conclusion

With 2014–2016 CFPS data, this paper found that medical insurance reimbursements for high-income people are higher than those for low-income people and that this difference is more significant in the NRCMI group than in the URBMI group. The study found that differences in health status are not the reason for inequity in medical insurance benefits; indeed, lower-income people have worse health. This paper further reveals the mechanism and finds that health care utilization and medical insurance reimbursement are the main causes of benefits inequity.

## Data Availability

The datasets used during the current study are not publicly available, but CFPS datasets can be accessed at Peking University Open Research dataverse: https://opendata.pku.edu.cn/dataverse/CFPS.

## Abbreviations

NRCMI: New rural cooperative medical insurance
URBMI: Urban residents’ basic medical insurance
CFPS: China family panel studies
SRH: Self-rated health
CNY: Chinese yuan

## References

[1] Bobo FT, Yesuf EA, Woldie M (2017). Inequities in utilization of reproductive and maternal health services in Ethiopia. Int J Equity Health.

[2] Zhu K, Zhang L, Yuan S, Zhang X, Zhang Z (2017). Health financing and integration of urban and rural residents’ basic medical insurance systems in China. Int J Equity Health.

[3] World Bank, 2004, Making services work for poor people, World Development Report.

[4] Davoodi HR, Tiongson ER, Asawanuchit SS (2012). Benefit incidence of public education and health spending worldwide: evidence from a new database. Poverty Public Pol.

[5] Wagstaff A, Bilger M, Buisman LR, Bredenkamp C. Who benefits from government health spending and why? A global assessment. 2014.

[6] Li YY, Zheng CR (2016). Benefit incidence of public medical service and its effects on income distribution: micro evidences based on household survey. Econ Res J.

[7] Cai J, Coyte PC, Zhao H. Decomposing the causes of socioeconomic-related health inequality among urban and rural populations in China: a new decomposition approach. Int J Equity Health. 2017;16(1). 10.1186/s12939-017-0624-9. 10.1186/s12939-017-0624-9 PMC5516311 28720105

[8] Wagstaff A, Yip W, Lindelow M, Hsiao WC (2009). China's health system and its reform: a review of recent studies. Health Econ.

[9] Zhou Q, Tian S, Pan J (2016). Inequity of equalization: theoretical and empirical analysis on beneficial equity of the urban resident basic medical Insurance in China. Econ Res J.

[10] Pan J, Lei X, Liu GG. Health insurance and health status: exploring the causal effect from a policy intervention. Health Econ, 2015, 25(11): 1389–1402. https://doi.org/10.1002/hec.3225. 10.1002/hec.3225 26350053

[11] Rawls J (1971). A theory of justice.

[12] Daniels N, Kennedy BP, Kawachi I (1999). Why justice is good for our health: the social determinants of health inequalities. Daedalus.

[13] Wagstaff A (2000). Research on equity, Poverty and Health Outcomes, Lessons from the Developing World.

[14] Yao Q, Liu C, Ferrier JA, Liu Z, Sun J. Urban-rural inequality regarding drug prescriptions in primary care facilities: a pre-post comparison of the National Essential Medicines Scheme of China. Int J Equity Health, 2015, 14(1). doi:10.1186/s12939-015-0186-7. 10.1186/s12939-015-0186-7 PMC4518678 26219841

[15] Pan J, Liu GG. The determinants of Chinese provincial government health expenditures: evidence from 2002–2006 data. Health Econ, 2011, 21(7): 757–777. https://doi.org/10.1002/hec.1742. 10.1002/hec.1742 21560182

[16] Wagstaff A (2002). Poverty and health sector inequalities. B World Health Organ.

[17] WHO (2000). The world health report 2000-Health systems: improving performance.

[18] Doorslaer EV, Wagstaff A, Bleichrodt H, Calonge S, Gerdtham UG, Gerfin M (1997). Income-related inequalities in health: some international comparisons. J Health Econ.

[19] Pannarunothai S, Mills A (1997). The poor pay more: health-related inequality in Thailand. Soc Sci Med.

[20] Gwatkin DR, Bhuiya A, Victora CG (2004). Making health systems more equitable. Lancet.

[21] Chu TB, Liu TC, Chen CS, Tsai YW, Chiu WT (2005). Household out-of-pocket medical expenditures and national health insurance in Taiwan: income and regional inequality. BMC Health Serv Res.

[22] Castro-Leal FJ, Dayton LD, Mehra K (2010). Public spending on health Care in Africa: do the poor benefit. B World Health Organ.

[23] Ping XQ (2003). The Seleetion of financing system of Medieal and health Service in the Rural Areas: a view based on the expending behavior in medical and health work for China peasants. Manage World.

[24] Wang SG, He HR, Le Y (2005). Policy-oriented, attraction and health equity. Chinese Soc Sci.

[25] Wei Z, Gustafsson B. Inequity in financing China’s healthcare. Econ Res J. 2005;(12):26–34 (In Chinese).

[26] Feng HB, Chen XJ (2009). An empirical study on the level of financial equalization of public medical and health expenditure. Financ Tr Econ.

[27] Xie E (2009). Income-related inequality of health and health care utilization. Econ Res J.

[28] Qi LS, Li ZN (2011). The income-related mobility of health and health care utilization. Econ Res J.

[29] Chen R, Li N, Liu X (2018). Study on the equity of medical services utilization for elderly enrolled in different basic social medical insurance systems in an underdeveloped city of Southwest China. Int J Equity Health.

[30] Zhou Z, Gao J, Fox A, Rao K, Xu K, Xu L (2011). Measuring the equity of inpatient utilization in Chinese rural areas. BMC Health Serv Res.

[31] Liu X, Gao W, Yan H (2014). Measuring and decomposing the inequality of maternal health services utilization in Western rural China. BMC Health Serv Res.

[32] Doorslaer EV, O’Donnell O, Rannan-Eliya RP, Somanathan A, Adhikari SR, Garg CC (2007). Catastrophic payments for health care in Asia. Health Econ.

[33] Wang Z, Chen Y, Pan T, Liu X, Hu H. The comparison of healthcare utilization inequity between URRBMI and NCMS in rural China. Int J Equity Health. 2019;18(1). 10.1186/s12939-019-0987-1. 10.1186/s12939-019-0987-1 PMC6567429 31200711

[34] Li XY (2009). Examining the equity of rural new cooperative medical and health system:health level, services utilization and fundraising. Chinese Popul Sci.

[35] Shen SG, Sun J, Liu Q, Zhou J (2009). A research on equity of new rural cooperative medical care system: take Guangdong Province for example. Popul Econ.

[36] Wong CK, Tang KL, Lo VI (2006). Unaffordable healthcare amid phenomenal growth: the case of healthcare protection in reform China. Int J Soc Welf.

[37] Duan NH, Manning WG, Morris CN, Newhouse JP (1983). A comparison of alternative models for the demand for medical care. J Bus Econ Stat.

[38] Liu YW, Wang RQ (2017). Income gap, social capital and Residents' poverty. J Quant Tech Econ.

[39] Yip WCM, Hsiao WC, Chen W, Hu S, Ma J, Maynard A (2012). Early appraisal of China’s huge and complex health-care reforms. Lancet.

[40] Whitehead M (1992). The concepts and principles of equity and health. Int J Health Serv.

